# Dealing with the unknown: perceptions and fears of SARS-CoV-2 infection among hospital workers

**DOI:** 10.1093/eurpub/ckac131.039

**Published:** 2022-10-25

**Authors:** M Utzet, R Villar, P Díaz, MP Rodríguez Arjona, JM Ramada, C Serra, FG Benavides

**Affiliations:** 1 Centre for Research in Occupational Health, Universitat Pompeu Fabra, Barcelona, Spain; 2 CIBER of Epidemiology and Public Health, Instituto de Salud Carlos III, Barcelona, Spain; 3 Hospital del Mar Medical Research Institute, Hospital del Mar, Barcelona, Spain; 4 Occupational Health Service, Parc de Salut Mar, Barcelona, Spain; 5 Funación Mémora, Barcelona, Spain

## Abstract

**Introduction:**

This study aims to explore perceptions, fears and worries regarding SARS-CoV-2 risk of infection and transmission to relatives and/or co-workers and non-pharmacological preventive interventions among hospital workers.

**Materials and Methods:**

The research used an explorative qualitative approach. Six focus groups and ten individual interviews across multidisciplinary workers (physicians, nurses, aides, cleaners, maintenance, and security staff) were conducted online and audio-recorded, transcribed verbatim and analysed using thematic analysis and mixed coding.

**Results:**

Forty professionals participated in the study. Four common themes emerged in all groups: challenges related to the lack of pandemic preparedness, concerns about personal protective equipment, unclear guidelines for case and contact tracing, and communication-related difficulties.

**Conclusions:**

This study emphasizes the key recommendations to improve non-pharmacological preventive interventions to reduce workers’ fears and worries about the risk of infection and spreading the infection to others, including families. Above all, these should include ensuring the availability, and correct use of adequate personal protective equipment, improve guidelines on case and contact tracing, and setting effective communication channels for all workers of the organization. These recommendations must be reinforced in maintenance and security personnel, as well as night shift nurses and aides, in order to reduce also health inequalities.

**Key messages:**

• Lack of pandemic preparedness increased HCW’ fear of infection, which could be reduced by ensuring the availability and good use of proper PPE, and by clear guidelines on case/contact detection.

• The improvement on non-pharmacological preventive interventions must be underpinned by effective communication channels and/or communication staff, and should reach all workers in the institution.

